# Diagnosis delay in Libyan female breast cancer

**DOI:** 10.1186/1756-0500-5-452

**Published:** 2012-08-21

**Authors:** Eramah Ermiah, Fathi Abdalla, Abdelbaset Buhmeida, Entesar Larbesh, Seppo Pyrhönen, Yrjö Collan

**Affiliations:** 1 Department of Oncology, University of Turku, Turku, Finland; 2 Department of Pathology, Turku University Hospital, Turku, Finland; 3 Department of Oncology, National Oncology Institute, Sabratha, Libya; 4 Department of Pathology, Misurata Cancer Institute, Misurata, Libya; 5 Center of Excellence in Genomic Medicine Research, King Abdul-Aziz University, Jeddah, Saudi Arabia; 6 Department of Pathology, National Oncology Institute, Sabratha, Libya

**Keywords:** Libyan female breast cancer, Diagnosis delay

## Abstract

**Aims:**

To study the diagnosis delay and its impact on stage of disease among women with breast cancer on Libya.

**Methods:**

200 women, aged 22 to 75 years with breast cancer diagnosed during 2008–2009 were interviewed about the period from the first symptoms to the final histological diagnosis of breast cancer. This period (diagnosis time) was categorized into 3 periods: <3 months, 3–6 months, and >6 months. If diagnosis time was longer than 3 months, the diagnosis was considered delayed (diagnosis delay). Consultation time was the time taken to visit the general practitioner after the first symptoms. Retrospective preclinical and clinical data were collected on a form (questionnaire) during an interview with each patient and from medical records.

**Results:**

The median of diagnosis time was 7.5 months. Only 30.0% of patients were diagnosed within 3 months after symptoms. 14% of patients were diagnosed within 3–6 months and 56% within a period longer than 6 months. A number of factors predicted diagnosis delay: Symptoms were not considered serious in 27% of patients. Alternative therapy (therapy not associated with cancer) was applied in 13.0% of the patients. Fear and shame prevented the visit to the doctor in 10% and 4.5% of patients, respectively. Inappropriate reassurance that the lump was benign was an important reason for prolongation of the diagnosis time. Diagnosis delay was associated with initial breast symptom(s) that did not include a lump (p < 0.0001), with women who did not report monthly self examination (p < 0.0001), with old age (p = 0.004), with illiteracy (p = 0.009), with history of benign fibrocystic disease (p = 0.029) and with women who had used oral contraceptive pills longer than 5 years (p = 0.043). At the time of diagnosis, the clinical stage distribution was as follows: 9.0% stage I, 25.5% stage II, 54.0% stage III and 11.5% stage IV.

Diagnosis delay was associated with bigger tumour size (p <0.0001), with positive lymph nodes (N2, N3; p < 0.0001), with high incidence of late clinical stages (p < 0.0001), and with metastatic disease (p < 0.0001).

**Conclusions:**

Diagnosis delay is very serious problem in Libya. Diagnosis delay was associated with complex interactions between several factors and with advanced stages. There is a need for improving breast cancer awareness and training of general practitioners to reduce breast cancer mortality by promoting early detection. The treatment guidelines should pay more attention to the early phases of breast cancer. Especially, guidelines for good practices in managing detectable of tumors are necessary.

## Background

In Libya, breast cancer is an important health problem among women. The incidence is 18.8 new cases per 100,000 women per year [1]. Most of the patients present with advanced disease [1,2]. The patients are often younger than in Europe, in line with the pattern common in Middle East and North Africa (MENA) [3].

To improve breast cancer care better understanding of the predicting factors and causes for treatment delay are important issues [4]. Small tumors are more likely to be treated successfully [5,6]. Delayed presentation of breast cancer is associated with advanced stage and low survival [7-9].

Studies in developed countries reported that median time to consultation was 14–61 days [10-12]. A delay of more than 3 months prior to physician consultation occurred in 14-53% of cases [12-15]. Low socio-economic status, minority ethnicity and young age were associated with a longer duration of symptoms [13]. Diagnosis delay was also associated with older age, lighter symptoms, fear of to informing anyone, negative attitude toward medical practitioners and fear of treatment [14].

Failures of medical practitioners to act on clinical findings, and false-negative mammogram or fine needle aspiration cytology (FNAC) were the main factors for delay after the visit to the general practitioner [15,16].

We conducted this study to learn more about the extent and reasons behind diagnosis delay of breast cancer in Libya.

## Patients and methods

The study group was two hundred Libyan female patients with breast cancer diagnosed at the African (presently (2012) National) Oncology Institute (NOI), Sabratha, during the period from Jan 1, 2008 to Dec 31, 2009. During that time 419 patients were registered at the institute. The patients were asked to be interviewed and the collection of data was stopped after 200 interviews had been completed.

### Data collection

Preclinical data was collected on a form (questionnaire) during the interview with each patient. Questionnaire for assessment of diagnosis delay is shown in Table 1.

**Table 1:** Questionnaire for assessment of diagnosis delay in women with breast cancer in Libya

**File number**		
Age (years)		
Address		
Occupation	House wife	
	Employee	
Marital status	Married	
	Single	
Education	Literate	
	Illiteracy	
Obstetric history	Age of menarche	
	Age at first pregnancy	
	Oral contraceptive pills	
	Number of births	
	Breast feeding	
Menopausal status	Pre-menopausal	
	Pos-menopausal	
Family history	Positive	
	Negative	
History of benign breast disease	Yes	
	No	
Breast self examination	Yes	
	No	
First symptoms	Lump	
	Nipple discharge	
	Skin changes	
	Systemic	
Mode of First symptoms detection (first symptoms)	Accidental	
	Breast self examination	
	Clinical examination	
Action taken by the patient at appearance of the first symptoms	Consult the doctor immediately	
	Delay	Duration of delay
		Reasons of delay
Action taken by the doctor at the first visit	Refer the patient	
	Medical follow-up	
	Duration of follow-up	
Duration between first symptoms and first visit to the doctor		
Duration between the first visit to the doctor and histopathological or cytological diagnosis		
Duration between the first symptoms and histopathological or cytological diagnosis.		
Clinical staging at time of the diagnosis (from the patients files)		

Structured face-to-face interviews were arranged either during the first hospitalization due to breast cancer (20%) or during follow-up in the outpatient department (80%). 22.5% of all interviews took place within 4 weeks after diagnosis and 77.5% within 8 weeks after diagnosis. The interviews were conducted by a trained physician (E.E and E.L) and required 30 to 45 minutes to complete.

The data collection included social and demographic data, medical and obstetric history, symptom-related questions, and consultation-related questions. Dates of the chronological events (first recognition of symptoms, first consultation, referral and first hospital appointment) were included. Diagnosis time and delays were estimated in days.

First symptoms included: lump, breast symptoms other than lump, and symptoms not related to the breast. The respondents were questioned about previous use of oral contraceptives, hormone replacement therapy or alternative therapy if these therapies were taken regularly for at least one month. Complementary alternative therapy included any therapy using methods and products not associated with conventional modern medicine.

In order to minimize recall bias, the respondents were reminded of events in the calendar year, such as religious and national occasions, school holidays and birth dates, to help them remember important dates relative to their medical history.

Data regarding tumour stage relied on histopathological and clinical data including TNM stage [17] were collected from medical records of each patient. A previous report suggests that the collection of data from medical records is more accurate than the patient interview alone [18].

### Diagnosis time

Diagnosis time was measured from the date of the first symptoms to the date of final breast cancer diagnosis based on histopathological examination (including needle biopsy or excisional biopsy) or on FNAC (fine needle aspiration cytology). Consultation time was the time taken to visit the general practitioner after the first symptoms.

### Statistical analysis

Diagnosis time was categorized into periods: <3 month, 3–6 months, and > 6 months and we use three months as cut-off point of delay [10,15]. Diagnosis was considered delayed if it took longer than 3 months after symptoms to reach the final diagnosis of breast cancer (diagnosis delay).

Socio-demographic characteristics, as potential determinants of diagnosis delay, included age, education, and employment status. Health characteristics, evaluated as potentially affecting the duration of diagnostic time, included menopausal status, use of oral contraceptives; breast self examination, history of fibrocystic disease, and family history of breast cancer.

Data were analyzed using SPSS for Windows (version 17, SPSS, Inc., Chicago, USA). The Chi-square test, with likelihood ratio (LR), or Fisher’s exact test was used to assess the significance of the association between potential predicator factors and diagnosis delay, and to identify independent determinants of diagnosis delay of 3 to 6 months and more than 6 months versus less than 3 month. Additionally the association between diagnosis delay and clinical stage was examined for all patients. In all tests, the values p <0.05 were regarded statistically significant.

### Ethical consideration

This study is a part of the breast cancer studies, which got permission from the local ethical committee of the African Oncology Institute (since 2012 the National Oncology Institute) Sabratha, Libya. All respondents in this study gave oral consent, after the character of the interview was explained to them. No patients refused the interview.

## Results

### Study population

The characteristics of the study population (n = 200) are shown in Table 2. The mean age of women was 45.4 years (range 22–75 years). Sixty-two percent of patients (n = 124) were literate. Seventy- nine percent of patients (n = 158) were married, multi-parous, and had fed their babies. 24.5% of patients (n = 49) had taken oral contraceptive pills, 26% did not have any children, and 36.5% were post-menopausal. Only 9% had a family history of breast cancer, 9.5% had a history of benign breast disease. In this study, 136 (68%) patients with breast carcinoma noted a lump or lumps as an accidental finding, while 4 (2%) patients detected lump(s) during self examination.

**Table 2:** Description of study population (n = 200)

		**Number of patients**	**Percent (%)**
Social demographic characteristics of the patients			
Age (years)	<50	133	66.5
	≥50	67	33.5
Education	Literate	124	62.0
	Illiterate	76	38.0
Occupation	House wife	111	55.5
	Employed	89	44.5
Marital status	Married	158	79.0
	Single	42	21.0
Medical history of the patients			
Menopausal status	Pre-menopausal	127	63.5
	Post-menopausal	73	36.5
Breast feeding	Yes	133	89.0
	No	16	11.0
Oral contraceptive	Yes <5 years	35	17.5
	Yes > 5 years	14	7.0
	No	151	75.5
Breast self examination	Yes	9	4.5
	No	191	95.5
Family history	Positive	18	9.0
	Negative	182	91.0
History of benign breast disease	Yes	19	9.5
	No	181	90.5
Clinical presentations of the patients			
Symptoms	Lump	136	68.0
	Nipple discharge	27	13.5
	Skin changes	31	15.5
	Systemic	6	3.0
Symptoms	Lump	136	68.0
	Others	64	32.0

Family physician noted a lump in 1 case and referred the patient to a proper health care facility. Other symptoms of the breast such as skin changes, nipple discharge or bleeding were reported less frequently (29%). Systemic involvements as the first symptom occurred in 6 (3%) patients.

### Diagnosis delay

**Figure 1:**
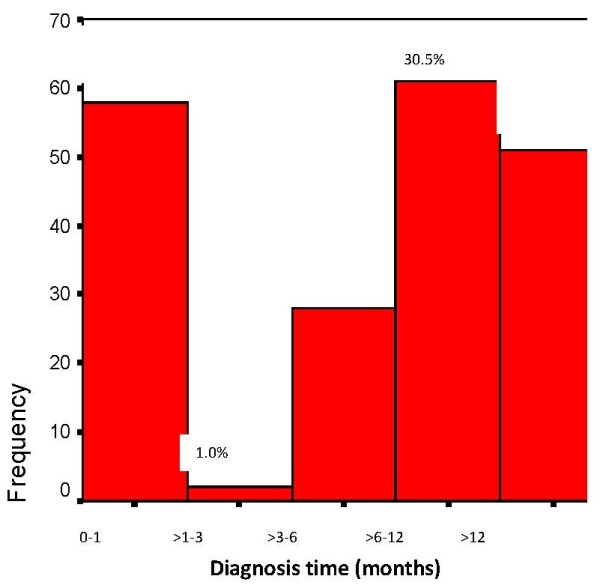
**Diagnosis time (from first symptoms) among 200 Libyan breast cancer patients (2008–2009).** Diagnosis time (months).

Diagnosis time is shown on Figure 1.

The median of diagnosis time was 7.5 months, 25 months as the maximum. 30% (n = 60) of patients were diagnosed within 3 months after detecting symptoms. 14% (n = 28) of patients were diagnosed within 3–6 months and 56% (n = 112) within a period longer than 6 months.

**Figure 2:**
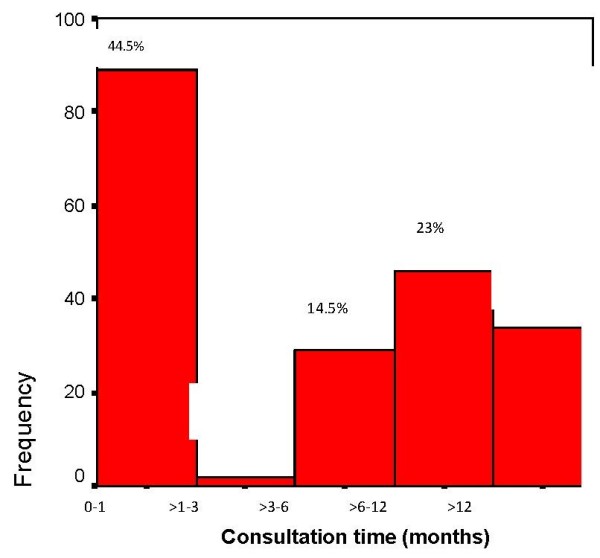
**Consultation time (from first symptoms) among 200 Libyan breast cancer patients (2008–2009).** Consultation time (months).

The median of consultation time was 4 months, 24 months as the maximum. 44.5% (n = 89) of patients had a medical consultation within one months after detecting symptoms, while 15.5% (n = 31) had visited the doctor within 1–6 months after symptoms. 40% (n = 80) of patients had consultation later than 6 months after first symptoms (Figure 2).

The majority of patients (84.5%; n = 169) were diagnosed within one month after the visit to the general practitioners. 4.5% of patients (n = 9) were diagnosed from 1 to 6 months after the first visit to the doctor, 22 (11.0%) patients had waited for more than 6 months for the final diagnosis after the first medical consultation.

### Clinical staging at time of diagnosis

At time of diagnosis, the clinical stage distribution was as follows: 9% stage I, 25.5% stage II, 54% stage III and 11.5% stage IV. The TNM staging is also shown in Table 3.

**Table 3:** Clinical staging and TNM classification at the diagnosis in 200 Libyan breast cancer patients 2008-2009

**Tumour characteristics**		**Number of patients**	**Percent (%)**
Clinical stage	Stage 1	18	9.0
	Stage 2	51	25.5
	Stage 3	108	54.0
	Stage 4	23	11.5
Clinical stage	Early stages (1 and 2)	69	34.5
	Late stages (3 and 4)	131	65.5
T	T 1	25	12.5
	T 2	54	27.0
	T 3	78	39.0
	T 4	43	21.5
N	N 0	50	25.0
	N 1	76	38.0
	N 2	58	29.0
	N 3	16	8.0
M	M 0	177	88.5
	M 1	23	11.5

### Diagnosis delay and associated factors

A number of factors predicted diagnosis delay: Symptoms were not considered serious in 54 (27%) patients. Alternative therapy was applied in 13.0% of the patients. Fear and shame prevented the visit to the doctor in 10% and 4.5%, respectively, of the patients. 31 patients (15.5%) were inappropriately reassured after first medical visit that the lump was benign.

Initial breast symptom(s) without a lump was strongly associated with diagnosis delay (p <0.0001). Diagnosis delay tended to be higher among women who did not report monthly breast self examination (p < 0.0001). Older women waited longer than younger women before presenting their symptoms to a physician (p *=* 0.004).

Additionally a significantly higher risk of delay was among illiterate (p = 0.009), among patients with a history of fibrocystic disease (p = 0.029) and among women who had used oral contraceptive pills longer than 5 years (p = 0.043). Diagnosis delay and associated factors are shown in Table 4.

**Table 4:** Diagnosis delay in Libyan breast cancer by an accordance to socio-economic factors, health behavior and tumor related factors

**Predicting factors**		**Number of patients**	**Proportion of patients according to diagnosis time (percent)**			**P value**
			**<3 months**	**3-6 months**	**>6 months**	
Age years	< 50	133	48.9	18.0	33.1	0.033
	≥ 50	67	35.8	11.9	52.2	
Age years	< 50	133	48.9	18.0	33.1	0.004
	50-65	32	53.1	12.5	34.4	
	≥ 65	35	20.0	11.4	68.6	
Current employment status	House wife	111	38.7	16.2	45.0	0.09
	Employed	89	51.7	15.7	32.6	
Education	Literate	124	61.6	18.1	20.3	0.009
	Illiteracy	76	30.9	15.8	53.3	
Menopausal status	Pre-menopausal	127	49.6	18.1	32.3	0.054
	Pos-menopausal	73	35.6	12.3	52.1	
Marital status	Married	158	43.7	14.6	41.8	0.648
	Single	42	47.6	21.4	31.0	
Breast feeding	Yes	133	41.4	13.5	45.1	0.09
	No	16	62.5	12.5	25.0	
Oral contraceptive	Yes <5 years	35	45.7	8.6	45.7	0.043
	Yes > 5 years	14	14.3	28.6	57.1	
	No t used	151	47.0	16.6	36.4	
Family history	Positive	18	55.6	5.6	38.9	0.324
	Negative	182	43.4	17.0	39.6	
History of benign breast disease	Yes	30	26.7	20.0	53.3	0.029
	No	170	47.6	15.3	37.1	
Breast self examination	Yes	9	100.0	0.0	0.0	<0.0001
	No	191	41.9	16.8	41.4	
Symptoms	Lump	136	58.8	16.9	24.3	<0.0001
	Others	64	14.1	14.1	71.9	

### Association between diagnosis delay and clinical staging

Late clinical stage of breast cancer was found in 65.5% of all patients (Table 3) and it tended to be more frequent among women with diagnosis delay >6 months (89.3%) than among women who had diagnosed < 3 months after onset of symptoms (23%; p < 0.0001).

Diagnosis delay was significantly associated with large tumour size (T3 and T4; p < 0.0001) and with positive lymph nodes (N2, N3; P < 0.0001).

23 patients presented with metastasis at time of diagnosis, 91.3% of those had diagnosis delay >6 months (p < 0.0001) after symptoms. Diagnosis delay and associated clinical stages of breast cancer at time of diagnosis are shown in Table 5.

**Table 5:** Diagnosis delay and risk of late stage breast cancer at time of diagnosis

**Tumour characteristics**		**Number of patients**	**Proportion of patients according to diagnosis time (percent)**			**P value**
			**<3 months**	**3-6 months**	**>6 months**	
Clinical stage	Stage 1	18	94.4	5.6	0.0	<0.0001
	Stage 2	51	74.5	23.5	2.0	
	Stage 3	108	2.8	10.2	87.0	
	Stage 4	23	0.0	0.0	100.0	
Clinical stage	Early stages	69	79.7	18.8	1.4	<0.0001
	Late stages	131	2.3	8.4	89.3	
T	T 1	25	96.0	4.0	0.0	<0.0001
	T 2	54	60.8	33.3	5.9	
	T 3	78	4.3	25.7	70.0	
	T 4	43	0.0	4.8	95.2	
N	N 0	50	80.0	18.0	2.0	<0.0001
	N 1	76	22.4	31.6	46.1	
	N 2	58	1.7	12.1	86.2	
	N 3	16	0.0	0.0	100.0	
M	M 0	177	32.8	21.5	45.8	<0.0001
	M 1	23	0.0	8.7	91.3	

## Discussion

Published data from the Middle East and North Africa (MENA) region suggest lower quality of health care among patients with cancer than in developed countries [18]. On other hand, the World Health Organization predicts an increase in cancer cases in developing countries. Significant advances in breast cancer management can probably improve the quality and efficacy of oncology practice in the MENA region [19,20].

This study shows the diagnosis delay of breast cancer is a serious problem in Libya. Through understanding the causes of delay it may be possible to reduce delays and to improve early diagnosis.

The average time before medical advice and diagnosis was long, and the diagnosis time was higher than in developed or developing countries [21]. Perhaps this trend can be attributed to low awareness of health issues among women, to poor information campaigns, and to the absence of mammographic or other screening programs for early detection of breast cancer in Libya.

This study showed that there was a relationship between the patient- associated factors delayed presentation of symptoms, in agreement with Ramirez et al. [7].

Patients are more likely to attribute new symptoms to less serious conditions than to life-threatening diseases [22]. Studies have reported delays when the patients assumed that symptoms were benign and would fade without interference [23].

We also observed that patients often considered symptoms as benign. This was the most important reason for delay in seeking doctors' advice as shown by Arndt et al. [10].

In current study, 13.0% of the respondents had taken alternative therapy, compared to 15-73% in Europe [24]. Most patients took alternative treatment as means to avoid surgery. Some patients believed that there were no effective treatments for breast cancer, or that traditional medicines are more effective than modern drugs.

While taking alternative treatments, most patients experienced worsening of symptoms, which eventually led to more advanced stage. The patients want to receive medical therapy but when the response is not favorable, they often use traditional medicine as the last hope [10].

This study showed that a negative information of breast cancer treatment caused delays as in Nigeria [25] and Malaysia [26]. Negative information, such as side-effects and expected toxicity of chemotherapy led to fear and refusal of therapy. Some patients believed that the effects of chemotherapy were worse than breast cancer itself. Fear of divorce or remarriage of the husband could lead some women to decide not to get their symptoms diagnosed if they suspected breast cancer. Some patients also believed that breast cancer could not be cured [27], so there was no point of having it diagnosed and treated. Diagnosis delay was also related to a belief that mastectomy causes disfigurement and disability [28].

There are certain ‘alarm symptoms’, important for create cancer diagnosis [29]. However, these are not always known within doctors or nurses. In the present study, we found that the respondents were inappropriately reassured after the first visit to the doctor that a lump can be considered benign without biopsy. This is a false attitude. In this study, this attitude was an important reason to the magnitude of the diagnosis time. Similar results were reported by Goodson et al. [16]. Even through the majority of lumps in young patients are benign, histological or cytological diagnosis should be available of every lump.

Interestingly, the present study revealed that the initial breast symptoms that did not include a lump were strongly associated with diagnosis delay. The discovery of a breast lump reduces the patient delay as confirmed in other studies [7,9]. The findings suggest that doctors and patients need to be educated about the different types of breast cancer symptoms.

Mammography is a sensitive means for early detection of breast cancer, but both clinical breast examination (CBE) and breast self-examination (BSE) have the potential to advance the diagnosis of breast cancer without the expense of a mammography facility [30]. Women with regular breast self-examination tended to seek medical care more rapidly and to have earlier stages of disease at diagnosis [31].

We observed that the patients who made self examination monthly were more educated, younger and asked for medical help rapidly than less educated or older patients.

Many studies have shown that older age is a predictor of diagnosis delay [7,20,32]. Since older age is a risk factor for both developing breast cancer and subsequent delayed presentation, any intervention program should target older women in particular [33]. In the current study, older women waited longer than younger women before presenting their symptoms to a physician.

The value of education on breast cancer symptoms has been reported in a number of studies [20,34]. Our findings suggest that lack of knowledge about breast cancer is an important factor in Libya and there is a need for public educational programs especially for less educated women. However, in Libya social values and moral considerations may limit the use of mass media for publicizing breast cancer awareness.

We observed that women with a history of fibrocystic disease were significantly associated with delay. The same results were reported by Arndt et al. [10]. Explanation is that former episodes of breast tissue alterations, if benign is also later considered as benign by their doctors.

Thus, it might be worthwhile to encourage women with known benign breast disease to present new breast symptoms promptly. Additionally, doctors should understand that new symptoms should be evaluated as potential new risks for breast cancer.

Similar to recent studies, our findings indicated that those patients who were diagnosed late had significantly bigger tumor size and presented with an advanced stage of the disease. The influence of delay on tumor size and disease stage is well documented [7,8].

Even through the present study does not provide information regarding the distribution of tumor differentiation, it is important to note that a substantial proportion of late stage diagnosis of poorly differentiated breast cancer cases could potentially have been avoided if patients with breast cancer had seen a doctor earlier [10].

## Conclusions

Diagnosis delay is very serious problems in Libya. Diagnosis delay was associated with complex interactions between social, medical and other patient-associated factors leading to advanced stages, potentially resulting in a high mortality. There is a need for improving breast cancer awareness and training of general practitioners to reduce breast cancer mortality by promoting early detection. The treatment guidelines should pay more attention to the early phases of breast cancer. Especially, guidelines for good practices in managing detectable of tumors are necessary.

## Competing interests

We (authors) declare that we have no conflict of interest.

## Authors’ contributions

E.E., performed research, designed the questionnaire and interviewed the patients; F.A., analyzed data statistically; E.L., collected the clinical data; A.B., S.P., and Y.C., designed and coordinated research and drafted the manuscript. All authors read and approved the final manuscript.
